# Association of Polygenetic Risk Scores Related to Immunity and Inflammation with Hyperthyroidism Risk and Interactions between the Polygenetic Scores and Dietary Factors in a Large Cohort

**DOI:** 10.1155/2021/7664641

**Published:** 2021-09-14

**Authors:** Mi Young Song, Sunmin Park

**Affiliations:** ^1^School of Food Science and Nutrition, Woo Song University, Daejeon, Republic of Korea; ^2^Department of Food and Nutrition, Obesity/Diabetes Research Center, Hoseo University, Asan 31499, Republic of Korea; ^3^Department of Bio-Convergence System, Hoseo University, Asan 31499, Republic of Korea; ^4^R&D, Yejun Bio, 165 Sechul-Ri, Baebang-Yup, Asan 31499, Republic of Korea

## Abstract

Graves's disease and thyroiditis induce hyperthyroidism, the causes of which remain unclear, although they are involved with genetic and environmental factors. We aimed to evaluate polygenetic variants for hyperthyroidism risk and their interaction with metabolic parameters and nutritional intakes in an urban hospital-based cohort. A genome-wide association study (GWAS) of participants with (cases; *n* = 842) and without (controls, *n* = 38,799) hyperthyroidism was used to identify and select genetic variants. In clinical and lifestyle interaction with PRS, 312 participants cured of hyperthyroidism were excluded. Single nucleotide polymorphisms (SNPs) associated with gene-gene interactions were selected by hyperthyroidism generalized multifactor dimensionality reduction. Polygenic risk scores (PRSs) were generated by summing the numbers of selected SNP risk alleles. The best gene-gene interaction model included tumor-necrosis factor (*TNF*)_rs1800610, mucin 22 (*MUC22*)_rs1304322089, tribbles pseudokinase 2 (*TRIB2*)_rs1881145, cytotoxic T-lymphocyte-associated antigen 4 (*CTLA4*)_rs231775, lipoma-preferred partner (*LPP*)_rs6780858, and human leukocyte antigen (*HLA*)-J_ rs767861647. The PRS of the best model was positively associated with hyperthyroidism risk by 1.939-fold (1.317–2.854) after adjusting for covariates. PRSs interacted with age, metabolic syndrome, and dietary inflammatory index (DII), while hyperthyroidism risk interacted with energy, calcium, seaweed, milk, and coffee intake (*P* < 0.05). The PRS impact on hyperthyroidism risk was observed in younger (<55 years) participants and adults without metabolic syndrome. PRSs were positively associated with hyperthyroidism risk in participants with low dietary intakes of energy (OR = 2.74), calcium (OR = 2.84), seaweed (OR = 3.43), milk (OR = 2.91), coffee (OR = 2.44), and DII (OR = 3.45). In conclusion, adults with high PRS involved in inflammation and immunity had a high hyperthyroidism risk exacerbated under low intakes of energy, calcium, seaweed, milk, or coffee. These results can be applied to personalized nutrition in a clinical setting.

## 1. Introduction

Hyperthyroidism is a condition that involves excessive productions of tetraiodothyronine (T4) and/or triiodothyronine (T3) by the thyroid gland and low serum levels of thyroid-stimulating hormone (0–0.4 mU/L; TSH) [1]. Graves' disease (an autoimmune disorder), Plummer's disease, and thyroiditis (thyroid gland inflammation) accompany hyperthyroidism [2]. The prevalence of Graves's disease and thyroid inflammation is linked to ethnicity [3]. Graves' disease prevalence is highest in Caucasians, followed by Hispanics, Africans, and Asians, but thyroiditis' prevalence follows the reverse order [4, 5]. The overall prevalence of hyperthyroidism was 0.5%, 0.7%, 1.2% in the USA, Europe, and Asia, respectively, during 1996–2018. Its global prevalence is higher in women than in men [4, 5]. In a cross-sectional Chinese study since 1995, the prevalence of hyperthyroidism among people living in iodine sufficient and insufficient areas were 1.2% and 1.0%, respectively (*P* < 0.001) [1], indicating that excess iodine and tetraiodothyronine intakes are involved in the induction of hyperthyroidism [1]. However, few studies have demonstrated an association between dietary intake and hyperthyroidism.

Hyperthyroidism contributes to digestive, cardiac, neural, and reproductive disorders [1]. Personal histories of certain chronic diseases, such as type 1 diabetes, adrenal insufficiency, and pernicious anemia, also act as risk factors of hyperthyroidism [5]. Furthermore, hyperthyroidism is associated with thyroid cancer, but it remains controversial. In the KoGES, the participants with either hyperthyroidism or hypothyroidism are positively associated with thyroid cancer risk [6]. Although thyroid cancer is linked to hyperthyroidism and hypothyroidism, their etiology is different, and their genetic association and interaction with lifestyles are different from thyroid cancer [6].

Hyperthyroidism has a high genetic predisposition of 70–80%, which was higher than environmental factors in twin studies [5, 7]. Previously reported studies have indicated that hyperthyroidism risk is associated with genetic variants of immunocompetent genes and inflammation-related genes associated with its etiology [7]. Genetic variants conferring hyperthyroidism risk include the human leukocyte antigen (HLA) complex and its gene family, composed of the *HLA-DRB1*, *HLA-DQA1*, *HLA-DQB1* genes [7]. In Korean children, HLA genetic variants are also associated with Graves' disease and Hashimoto's disease [8]. More specifically, genetic risk factors of Graves' disease accompany having the risk alleles of HLA-DR3, a cluster of differentiation 152 (CD152 or CTLA4), protein tyrosine phosphatase nonreceptor type 22 (*PTPN22*), CD40, interleukin (IL)-2 receptor alpha chain (*IL2RA*), IL-23 receptor (*IL23R*), and Fc receptor-like 3 (*FCRL3*) [7]. Some ethnic differences in genetic predisposition have been demonstrated by meta-analysis [9, 10]. The tumor necrosis factor-alpha (*TNF-α*) rs1800629 polymorphism was reported to exhibit a 1.97-fold association with hyperthyroidism (95% confidence intervals = 1.27–3.06, *P*=0.002) in 2,790 Graves' disease patients and 3,472 healthy controls, but subgroup analysis revealed a genetic impact on Europeans and not in Asians [11]. A meta-analysis demonstrates that *TNF-α* at 308 G/A and IL-6 at 174 G/C polymorphisms exhibit increased hyperthyroidism risk in Caucasians. However, Asians show different genetic polymorphisms for hyperthyroidism risk: *IL-1α* at 889 C/T, *IL-1β* at 511 C/T, *IL-6* at 174 G/C, *IL-6* at 572 G/C, and *IL-10* at 1,082 A/*G* polymorphisms [10].

Although genetic impacts and lifestyles are known to influence the risk of hyperthyroidism, no study has yet addressed the effects of their interactions. This study tested the hypothesis that polygenetic variants involved in inflammation and immunity are associated with hyperthyroidism risk and interact with metabolic parameters and nutritional intakes. This hypothesis was evaluated in adult participants aged >40 of a hospital-based urban cohort from 2004–2013, a part of the Korean Genome and Epidemiology Study (KoGES).

## 2. Materials and Methods

### 2.1. Participants and Hyperthyroidism Criteria

Korean middle-aged and elderly adults (age >40 years, *n* = 58,645) voluntarily participated in the KoGES study during 2004–2013, which was organized by the Korean Center for Disease and Control and approved by the Institutional Review Boards of the Korean National Institute of Health (KBP-2015-055) and Hoseo University (1041231-150811-HR-034-01). All participants provided informed consent in writing.

Participants who were previously diagnosed with hyperthyroidism by a physician were considered as cases (hyperthyroidism), while the rest were designated as controls. Hyperthyroidism is involved in Graves' disease, subacute thyroiditis, silent thyroiditis, and autonomous functional thyroid nodules, but the disease related to hyperthyroidism was not specified in the KoGES. However, 18,716 participants had not been examined for diagnosis of hyperthyroidism, and 288 participants did not answer the hyperthyroidism diagnosis question. These 19,004 participants were eliminated from the analysis. After these exclusions, 39,641 participants (842 cases and 38,799 controls) constituted for genetic-related analysis in the present study cohort. Among the participants with hyperthyroidism history, 312, 276, and 254 participants (37.0, 32.8, and 30.2% among the participants having hyperthyroidism) were completely cured, taking medication, and no treatment, respectively. All 842 participants with hyperthyroidism history were included in the genetic analysis since the genetics were not changed regardless of treatment and complete remission. However, the 312 participants with complete remission were not included in the statistical analysis of clinical and lifestyle parameters, including nutrient intake, since lifestyles interacted with hyperthyroidism.

### 2.2. General Characteristics and Anthropometric and Biochemical Measurements

General personal data, including age, education, income, smoking history, alcohol consumption, and physical exercise, were surveyed with a health interview [12]. Education and income level were divided into three groups as previously described [13]. Smoking status was categorized into current smoker, past smoker, and never-smoker, according to more than 100 cigarettes last six months [13]. Alcohol consumption was calculated by frequency and drinking amounts at a time (g/day), and the participants were categorized into nondrinker (0), mild drinker (0–20), and moderate drinker (>20) (Table 1) [13].

All participants had over 12–16 h fasting for food and drink, and the anthropometry and biochemical measurement proceeded. A skilled technician measured each participant's body weight, height, and waist circumferences according to a standardized procedure [14]. Body mass index (BMI) was calculated by dividing weight in kilograms by height in meters squared. Plasma and serum samples were collected for biochemical analysis [14]. Serum total cholesterol, HDL, triglyceride, and plasm glucose concentrations were assessed using a Hitachi 7600 Automatic Analyzer (Hitachi, Tokyo, Japan). White blood cell (WBC) counts were obtained using EDTA-treated blood. Blood pressures were measured on right arms at heart height in a sitting position three times, and average values were used for blood pressure.

### 2.3. Daily Nutrient Intake and Dietary Pattern Analysis

Usual dietary intake during the last year was estimated using a semiquantitative food frequency questionnaire (SQFFQ) developed and validated by the committee of KoGES. An SQFFQ validated for KOGES was used [15]. 56,934 participants completed this questionnaire, which requested consumption information regarding frequencies and amounts of 106 food items with assigned serving sizes. The intake of 23 nutrients were estimated using a Computer-Aided Nutritional Analysis Program 3.0 developed by the Korean Nutrition Society [15].

The 106 food items were categorized into 29 food groups used as independent variables in a factor analysis to classify dietary patterns. The number of factors was determined using eigenvalues of >1.5 in principle component analysis, and the orthogonal rotation procedure (Varimax) was applied to determine dietary patterns [16]. Dietary factor-loading values of ≥0.40 were accepted as an indication of significant contributions to dietary patterns.

### 2.4. Dietary Inflammatory Index (DII)

The DII was compiled utilizing the dietary inflammatory weightings of foods and nutrients (energy, 32 nutrients, 4 food products, 4 spices, and caffeine). Literature-based dietary inflammatory weightings were used [17]. Since intake of spices was not included in the SQFFQ, garlic, ginger, saffron, and turmeric intakes were excluded from DII calculations, which were performed by multiplying the dietary inflammatory weights of the 38 individual dietary components by their daily intakes and dividing the sum of these food items by 100.

### 2.5. Quality Control of Genotyping

Genetic variant data were received from the Center for Genome Science at the Korea National Institute of Health. Genomic DNA was separated from whole blood and genotyped using a Korean Chip (Affymetrix, Santa Clara, CA). This chip included known disease-related single nucleotide polymorphisms (SNPs) and was developed to study associations between Korean genetic variants and diseases [18]. Bayesian Robust Linear Modeling combined with the Mahalanobis Distance Genotyping Algorithm was used to assess genotype accuracy [19]. The accepted DNA samples conformed with the following criteria: genotyping accuracy (≥98%), missing genotype call rates (<4%), repeated heterozygosity (<30%), or no gender biases. Genetic variants were also required to satisfy HWE inclusion criteria (*P* > 0.05) [15].

### 2.6. Generation of the Best Model for Gene-Gene Interactions by Generalized Multifactor Dimensionality Reduction (GMDR)

**Figure 1:**
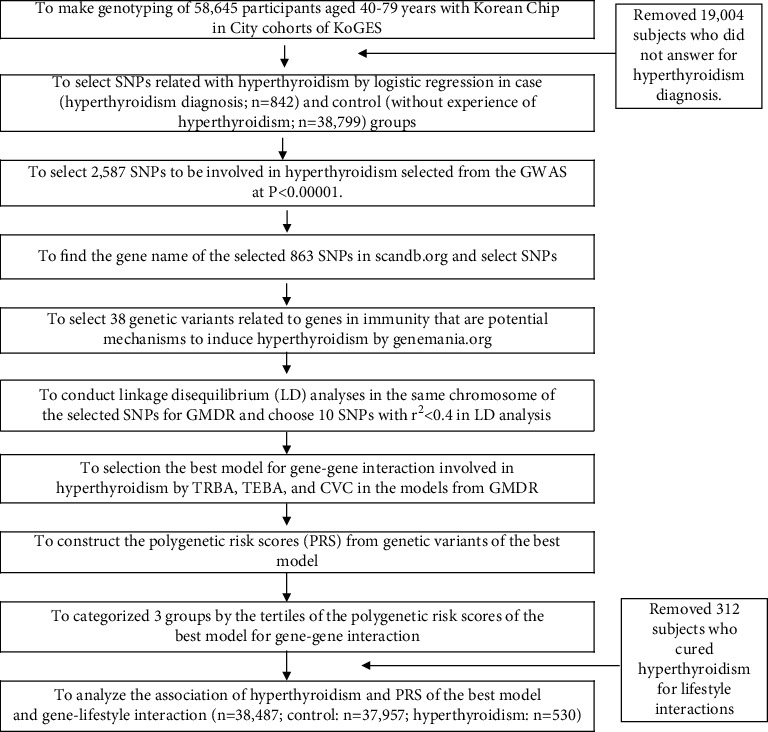
Flow chart to generate polygenetic risk score system influencing hyperthyroidism risk.

Figure 1 illustrates the method used to determine polygenetic risk scores (PRSs) for the hyperthyroidism risk. 1. Participants were dichotomized into cases (*n* = 842) and controls (*n* = 38,799). A genome-wide association study (GWAS) using PLINK version 2.0 (http://pngu.mgh.harvard.edu/∼purcell/plink) was used to identify genetic variants associated with hyperthyroidism risk, and genetic variants were accepted under the *P* < 0.00001.2,587 genetic variants were accepted, and the corresponding gene names were identified using scandb.org. The 863 genetic variants without corresponding gene names were removed. Genes corresponding to the selected SNPs for hyperthyroidism risk were screened for “inflammation” and “thyroid”. The 38 SNPs selected were subsequently checked for linkage disequilibrium (LD) with selected genetic variants in the same chromosomes using LocusZoom (http://genome.sph.umich.edu/wiki/LocusZoom _Standalone). Those with strong LDs were removed since they provided similar information concerning the risk of hyperthyroidism. Finally, ten potential genetic variants were accepted for constructing the best model.

The best gene-gene interaction model for hyperthyroidism risk was evaluated by trained balanced accuracy (TRBA), test balance accuracy (TEBA), and crossvalidation consistency (CVC) using GMDR analysis [17]. The best gene-gene interaction model was chosen in the GMDR test by the sign rank test of TRBA and TEBA values with covariate adjustments for age, gender, residence area, education, family income, and BMI. The statistical significance in the sign test was determined with a *P* value <0.05. CVC was checked by 10-fold crossvalidation since the sample size exceeded 1000 [17]. The risk allele of each best model SNP was counted as 1, and PRSs were calculated as the sum of the risk allele scores of each SNP in the best model [20]. For example, if the C allele was associated with a higher risk of hyperthyroidism, TT, CT, and CC were assigned 0, 1, and 2. Best models with 6 or 7 SNPs were categorized as (0–3, 4–6, and ≥7) and (0–4, 5–7, and ≥8), respectively. Each group was designated as the low, medium, and high PRS group.

### 2.7. Statistical Analyses

Statistical analyses were conducted using Plink and SAS version 9.3 (SAS Institute, Cary, NC, USA). A descriptive statistics for categorical variables (e.g., gender and lifestyle) were calculated based on frequency distributions by three PRS groups. Chi-squared tests were used for assessing frequency distributions of categorical variables. Adjusted means and standard errors were calculated for continuous variables based on the presence or absence of hyperthyroidism. Statistical differences between case and control groups were conducted using the analysis of covariance (ANCOVA) after adjusting for covariates. Adjusted ORs and 95% confidence intervals for hyperthyroidism according to the three groups of PRSs were determined by multiple regression analysis after covariate adjustment. Participants were separated into high and low dietary intake groups to investigate interactions among PRS and dietary intake parameters. Two-way ANCOVA with main effects and an interaction term was used to investigate interactions between PRS and lifestyle parameters after adjusting for covariates. *P* values < 0.05 were considered to be statistically significant.

## 3. Results

### 3.1. General, Anthropometry, and Biochemical Characteristics of Participants with Hyperthyroidism

The adjusted mean age of hyperthyroidism (*n* = 530) was higher than that of controls (*n* = 38,799), but age was not associated with hyperthyroidism risk (Table 2). Covariates included gender, age, area of residence, surveyed year, body mass index (BMI), smoking, alcohol, education, job, income, energy intake, arthritis, and dermatitis medicine intake. The average age of hyperthyroidism diagnosis was 47.7 years. Women had a 3-fold higher risk of hyperthyroidism than men (Table 2). Adjusted means of BMI and serum total cholesterol concentrations were small but significantly lower for cases than controls (Table 2). No significant association was found between metabolic syndrome (MetS) or its components and hyperthyroidism risk. Thyroid cancer had a much higher prevalence among cases than controls, and hyperthyroidism risk was 2.9-fold higher in participants with thyroid cancer. Adjusted mean serum high-sensitivity C-reactive protein (hs-CRP) concentration was greater in cases than controls, but inflammation index, white blood cell (WBC) count, and serum hs-CRP concentrations were similar (Table 2). Differences in the education or income status between cases and controls were not significant, and education and income status exhibited no significant associations with hyperthyroidism risk after the adjustment for covariates (gender, age, residence area, surveyed year, BMI, smoking, alcohol, education, job, income, energy intake, arthritis, and dermatitis medicine intake) (Table 2).

### 3.2. Lifestyles and Nutrient Intakes

Since nutrient intake differences were analyzed between hyperthyroidism and control, the participants who had complete remission for hyperthyroidism on the survey day were excluded for nutrient analysis (hyperthyroidism group: *n* = 530; control group: *n* = 38,799). After adjusting for designated covariates, energy intakes were similar in the hyperthyroidism and control groups (Table 1). Carbohydrate intake was higher, and fat intake was lower in the hyperthyroidism group, and significant intergroup differences were not observed for protein, fiber, Ca, or Na intakes (Table 1). Seaweed and vitamin C intakes and dietary inflammatory index (DII) were also not significantly different between the control and hyperthyroidism groups. Korean balanced dietary (KBD) and rice-based diet (RBD) categorized by principal component analysis did not differ between the two groups (Table 1). KBD included beans, potatoes, kimchi, vegetables, fish, chicken, milk, fruits, and tea. Western-style diet (WSD) was composed of eggs, processed meat, noodles, soups, and RBD, mainly rice (Supplemental Table 1). However, participants with a high WSD intake had a lower prevalence of hyperthyroidism than controls (Table 1); and the risk of hyperthyroidism was 0.818-fold lower for participants with a high WSD intake (Table 1). Alcohol and coffee intakes differed significantly between the case and control groups, but no significant association with hyperthyroidism was evident (Table 1). Furthermore, daily regular exercise and alcohol consumption were not significantly associated with hyperthyroidism risk after covariate adjustments (Table 2).

### 3.3. Genetic Variants Associated with Hyperthyroidism Risk and Gene-Gene Interactions between the Genetic Variants by GMDR

For genetic variants associated with hyperthyroidism risk by a genome-wide association study (GWAS), we selected genetic variants exhibiting gene-gene interactions using GMDR. Ten genetic variants involved in autoimmunity and inflammation were utilized in the GMDR analysis. The genetic characteristics of the 10 SNPs are shown in Table 3. Seven SNPs were positively (OR>1) and three were negatively associated with hyperthyroidism risk (0<OR<1). Seven SNPs were located in chromosome 6 (6p21) and were involved in inflammation and autoimmunity (Table 3). For all SNPs, the *P* value of the Hardy–Weinberg equilibrium (HWE) was >0.05, indicating all met the HWE criterion (*P* > 0.05). These SNPs were not associated with thyroid cancer.

After conducting GMDR, the best gene-gene interaction models included 6 and 7 genetic variants. The best model with 6 SNPs included microRNA 36891 (*MIR3681*), cytotoxic T-lymphocyte-associated antigen 4 (*CTLA4*), lipoma-preferred partner (*LPP*), mucin 22 (*MUC22*), tumor necrosis factor (*TNF*), and major histocompatibility complex, class I, J (Pseudogene; *HLA-J*) after adjusting for age and gender (adjustment 1) or age, gender, survey year, residence area, and BMI (adjustment 2, Table 4). The best 7-SNP model included the 6 SNPs in the 6-SNP model plus testis-expressed basic protein 1 (*TSBP1*). These 6- and 7-SNP models had trained balanced accuracy (TRBA) of 0.5888 and 0.6172, and a test balance accuracy (TEBA) of 0.5221 and 0.5193, respectively, after adjusting for age, gender, seaweed intake, and BMI (adjustment 2, *P* < 0.001). Crossvalidation consistency (CVC) for both models was 10/10 (Table 4). These results indicated that the 6- and 7-SNP models exhibited gene-gene interactions that influenced the genetic risk of hyperthyroidism.

### 3.4. Association between Polygenetic Risk Scores (PRSs) and Hyperthyroidism Risk

**Figure 2:**
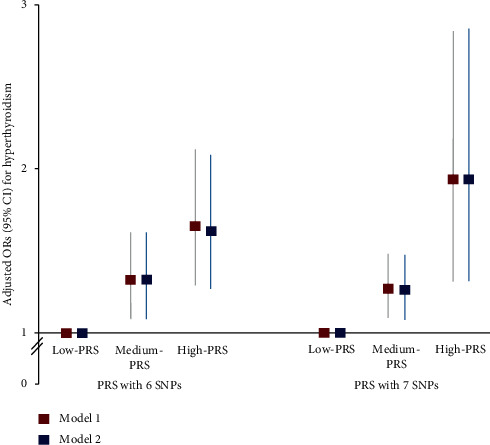
Adjusted odds ratio (ORs) and 95% confidence intervals (CIs) of the PRSs of 5- and 6-SNP models generated assessing gene-gene interactions associated with hyperthyroidism risk. The best GMDR models with 6 SNPs and 7 SNPs were calculated by the summation of the number of risk alleles of six and seven SNPs, and the calculated PRSs were divided into three categories (0–3, 4–6, and ≥7) and (0–4, 5–7, and ≥8), respectively, as the low PRS, medium PRS, and high PRS groups. The adjusted OR was analyzed by logistic regression with the covariates including age, gender, residence areas, initial menstruation age, menopause, pregnancy experience, income, education, energy intake, seaweed intake, smoking status, physical activity, WBC counts, alcohol intake, autoimmune diseases, and survey year. The reference group was the low PRS in logistic regression. Red and blue boxes indicated the adjusted ORs for five SNPs and six SNPs, respectively, and the lines through red and blue boxes indicated 95% CIs.

The PRS was constructed from the 6- or 7-SNP model among the GMDR models. PRSs were categorized into three groups (low, medium, and high PRS). In the 6- and 7-SNP models, a high PRS increased hyperthyroidism risk by 1.62- and 1.94-fold, respectively, compared with a low PRS (Figure 2). The 6- and 7-SNP models were subjected to two adjustments to include different covariates. Adjustment 1 included age, gender, residence area, survey year, BMI, education, job, and income as covariates, and adjustment 2 included age, gender, residence area, survey year, smoking, alcohol, education, job, income, energy, activity, hypertension, milk, percent fat intake, percent carbohydrate percent intake, and arthritis and dermatitis medicine intakes. Because the 7-SNP model had a higher adjusted odds ratio (ORs) for hyperthyroidism risk than the 6-SNP model, it was used for further analysis. Notably, no significant association was observed between MetS or its components and PRS calculated using the 6- or 7-SNP models (data not shown), indicating that PRS obtained using the 6- and 7-SNP models were not uniquely associated with hyperthyroidism.

### 3.5. Genetic Interactions of Lifestyles with Hyperthyroidism Risk

The interaction between age and PRS from the 7-SNP model influenced hyperthyroidism risk. A high PRS indicated a much greater risk of hyperthyroidism than a low PRS in participants aged <55 years but not in participants aged ≥55 years (Table 5). The prevalence of hyperthyroidism was much greater in participants <55 years old with a high PRS than a low PRS (Supplemental Fig.  S1A). However, no interaction was observed between gender and PRS (*P*=0.31). Interestingly, PRS interacted with hyperthyroidism risk (*P*=0.017): PRS was positively associated with hyperthyroidism risk only in the participants without MetS (OR = 2.28, *P* < 0.001; Table 5) but not in the participants with MS (Table 5). The hyperthyroidism frequencies were much higher in the high PRS than the low PRS, only in the participants without MetS, but there was no difference in hyperthyroidism incidence among the PRS groups in the participants with MetS (Supplemental Fig.  S1B). These results suggest that MetS offsets the genetic impact on hyperthyroidism risk. The BMI and PRS did not interact to influence hyperthyroidism risk (Table 5).

The interaction between energy intake and PRS affected hyperthyroidism risk (*P*=0.008; Table 5). Among participants with a low energy intake, the prevalence of hyperthyroidism was much higher in those with a high PRS than a low PRS, but no significant energy intake × PRS interaction was observed among participants (Supplemental Fig.  S1C). Furthermore, no interactions were found for carbohydrate (*P*=0.448), protein (*P*=0.429), fat (*P*=0.097), or fiber (*P*=0.707) intakes. However, daily Ca intake interacted with PRS (*P*=0.013). Among participants, a high PRS was positively associated with hyperthyroidism risk only in those with a low Ca intake (Supplemental Fig.  S1D). On the other hand, food, fruit, vegetable, and alcohol intakes did not interact with PRS (Table 5), but interestingly, seaweed (*P*=0.020), milk (*P* < 0.0001), and coffee (*P*=0.019) intakes did interact with PRS (Table 5). The participants with a low PRS had a much higher prevalence of hyperthyroidism than those with a high PRS and low intake of milk, seaweed, or coffee (Supplemental Fig.  S1E–Fig.  S1G). DII scores were also found to interact with PRS to influence hyperthyroidism risk (*P*=0.0499). Among participants with low DII scores, the prevalence of hyperthyroidism was much higher in those with a high PRS than a low PRS, but no significant difference was observed among participants with high or low DII scores (≥75th percentile; Supplemental Fig.  S1H). Daily regular exercise and smoking status showed no interaction with PRS (Table 5).

A KBD pattern included the consumption of beans, potatoes, kimchi, green and white vegetables, mushrooms, fatty and white fish, seaweeds, fruits, and pickles (loading ≥0.4). Participants with a WSD pattern preferentially consumed meat, noodles, soups, and fast foods, and those with an RBD pattern consumed mainly rice-based dishes (Supplemental Table 1). KBD, WSD, and RBD patterns showed no interaction with PRS for hyperthyroidism risk (Table 5).

## 4. Discussion

Hyperthyroidism is associated with a genetic predisposition in >70% of cases, more than observed in any other metabolic disease [5]. Hyperthyroidism is a polygenetic disease and is prevalent in genetic variants related to the immune system and inflammation. For example, genetic variants of *TNF-α*, *IL-1*, *IL-6*, and *IL-10* have been reported to increase the risk of hyperthyroidism [9, 11]. However, the risks of hyperthyroidism posed by lifestyles, such as food intake, have not been well-studied, though it has been established that seaweed intake is positively associated with hyperthyroidism risk when the thyroid gland cannot adapt to excess iodine intake [21]. However, the relation between seaweed consumption and the risk of hyperthyroidism remains unclear [22], and the influence of interactions among genetic variants and dietary and lifestyle factors on hyperthyroidism risk have not been examined. We hypothesized that polygenetic variants of genes involved in inflammation and immunity are associated with hyperthyroidism risk and interacted with metabolic parameters and nutritional intakes to modulate the risk of hyperthyroidism. This hypothesis was evaluated in 39,641 individuals aged >40 (847 had hyperthyroidism) who participated in the urban hospital-based cohort (2004–2013). The present study is the first study to demonstrate that PRSs derived from genes associated with inflammation and immunity interact with MetS parameters and food intake to modulate the risk of hyperthyroidism.

The most prevalent causes of hyperthyroidism in Korea in decreasing order are Graves' disease (82.7%), subacute thyroiditis (13.3%), painless thyroiditis (3.5%), and toxic adenoma (0.5%) [23]. Therefore, most hyperthyroidism might be related to Graves' disease in the present study. The primary cause of hyperthyroidism was Graves' disease, an autoimmune disease associated with high levels of antibodies to the TSH receptor and manifesting as low serum levels of TSH, stimulating the thyroid gland to produce T3 and T4 [23]. Thyroiditis is related to viral attack or antibody production to thyroid antigen via CD4 Th1 response, which results in progressive destruction of the thyroid gland [24]. Thus, autoimmune responses are the potential cause of hyperthyroidism. Autoimmune diseases such as allergies, arthritis, and asthma were used as covariates in the present study.

Some genetic variants that increase the risk of hyperthyroidism have been reported to be related to immunity and inflammation. *TRIB2* rs1881145 has been associated with Graves' disease in a Chinese study [25]. Furthermore, the *CTLA4* gene has a critical immunomodulatory function in maintaining peripheral self-tolerance, and the *CTLA4* gene variants +49 A/*G* and CT 60 A/*G* were found to be associated with Graves' disease in a Kashmiri population [26]. The present study also showed that *CTLA4* rs231775 is associated with hyperthyroidism. A few studies have investigated *HLA-J* and established that its expression is elevated in breast cancer biopsies and that this is associated with the overexpression of estrogen receptor 1 (*ESR1*), which has immunosuppressive activities [27]. In addition, the overexpression of *HLA-J* after neoadjuvant chemotherapy has been reported to be associated with reduced survival rates in breast cancer [27], which suggests altered immune evasion caused by *HLA-J* rs767861647 mutation might be involved in breast cancer progression. Furthermore, several genes, including TNF, mucin 22 (*MUC22*), testis-expressed basic protein 1 (*TSBP1*), transporter 2, ATP binding cassette subfamily B member (*TAP2*), and inositol 1,4,5-trisphosphate receptor type 3 (*ITPR3*) located near the *HLA-J* gene in chromosome 6 were found to be related to hyperthyroidism risk in the present study. *TAP2* is involved in defective major histocompatibility complex (MHC) class I expression and antigen presentation in autoimmune diseases, such as celiac disease and type 1 diabetes [28]. TNF has been previously reported to be related to hyperthyroidism. In patients with Graves' disease, serum TNF receptor protein levels were positively correlated with serum T3 and T4 concentrations and were negatively correlated with serum TSH concentrations [29]. Anti-TNF therapy reduces free T4 concentrations in Graves' disease patients [30]. Therefore, the genes selected in the present study showed the potential to increase hyperthyroidism risk by modulating immunity and inflammation.

Negative feedback regulates thyroid hormone secretion through hypothalamus⟶anterior pituitary gland⟶thyroid gland axis, and thyrotropin-releasing hormone secreted by the hypothalamus stimulates a TSH release from the anterior pituitary and stimulates thyroid hormone release [31]. TSH is the main stimulator of thyroid hormone secretion, as determined by measuring thyroid hormone's blood concentrations [31], a primary regulatory hormone of energy metabolism. Furthermore, serum TSH concentrations are reportedly associated with weight gain and MetS [32]. Although thyroid dysfunction commonly enhances MetS risk [31, 33], most investigations of the relationship between thyroid dysfunction and MetS have focused on hypothyroidism. Subclinical and clinical hypothyroidisms have been demonstrated to be associated with MetS risk and increased insulin resistance [34], and hyperthyroidism has also been reported to increase the risk of insulin resistance and hyperglycemia [34]. However, we did not find an association between hyperthyroidism and MetS risk or any component of MetS in Korean adults aged >40. Nonetheless, the present study demonstrates that patients with hyperthyroidism are at higher risk of thyroid cancer (OR = 2.91) and that 7.8% of thyroid cancer patients had hyperthyroidism, which concurs with previous results, which reported 1.6–21.1% of thyroid cancer patients have hyperthyroidism [35]. These results caution that clinicians should be aware of the risk of thyroid cancer in hyperthyroidism patients.

The present study showed that genetic variants involved in immunity and inflammation interact with dietary intake to modulate the risk of hyperthyroidism. Although dietary intake itself was not associated with hyperthyroidism risk, energy, Ca, milk, coffee, and seaweed intakes interacted with PRS and influenced hyperthyroidism risk. More specifically, only participants with a high PRS that consumed low amounts of energy, Ca, milk, coffee, and seaweed had a higher risk of hyperthyroidism. Furthermore, participants with a low DII and a high PRS were at higher risk of hyperthyroidism than those with a low PRS. In a previous study, inflammation and oxidative stress are associated with thyroid dysfunction [36]. However, studies on relationships between lifestyle-related variables and hyperthyroidism risk are limited [37], as are studies on relationships between diet and hyperthyroidism, other than for seaweeds and iodine.

Thyroid hormone contains iodine, and the relationship between iodine intake and hyperthyroidism has been well-studied. However, unlike hypothyroidism, the association between iodine intake and hyperthyroidism remains equivocal. Koukkou et al. concluded that excessive iodine intake might contribute to excessive thyroid hormone synthesis and release, inducing autonomic thyroid function and increasing the risk of iodine-induced hyperthyroidism in those living in abundant iodine areas [37]. Bajuk et al. have reported that high iodine intake reduced the incidence of iodine-induced hyperthyroidism in Slovakians (*P* < 0.001) [38]. Park et al. have demonstrated that excessive iodine intake did not affect hyperthyroidism in a Korean cohort [39]. In a longitudinal study, low iodine intake tended to increase the risk of hyperthyroidism in adults at a young age, despite subsequent sufficient iodine intake [40]. These results suggest that excessive iodine intake does not stimulate hyperthyroidism and that iodine restriction diets may negatively affect the management of hyperthyroidism. In the current study, seaweed intakes were similar in cases and controls, but participants with a high PRS and low seaweed intake had a higher rate of hyperthyroidism than those with a low PRS in low seaweed intake, though the same was not observed for high seaweed intake. These observations suggest that high iodine intake (>2.65 g seaweed/day) does not increase hyperthyroidism risk, especially in individuals with a high PRS.

The present study has several limitations that warrant consideration. First, the study was conducted using a case-control design, and, thus, we cannot comment on the causality of the effects observed. Second, the history of hyperthyroidism diagnosed by physicians was used to set hyperthyroidism criteria, and some control subjects might not be diagnosed yet, although they may have hyperthyroidism. Third, since the diagnosis of hyperthyroidism risk was used as a case criterion, thyroid disease types, such as Graves' disease and thyroiditis, were not specified. Fourth, serum T3 and T4 concentrations were not measured. Last, food intakes may have been inaccurate because they were quantified with semiquantitative food frequency questionnaire (SQFFQ) responses, which are known to have limitations in determining usual food intakes. Nevertheless, the SQFFQ used for this study was designed and validated for KoGES. The usual food intake was measured during the previous year, and the dietary intake was not directly related to hyperthyroidism risk.

## 5. Conclusion

Women with thyroid cancer history were at about a 3-fold risk of hyperthyroidism than men and those with no thyroid cancer experience. Carbohydrate intake was positively associated with hyperthyroidism risk, whereas WSD and alcohol intake were negatively associated. Inflammation and immunity-related genetic variants exhibited SNP-SNP associations with hyperthyroidism risk. Furthermore, the PRS was found to interact with age, MetS, and dietary intake. These results suggest that hyperthyroidism is associated with genetic factors that impact inflammation and immunity and that lower intakes of energy, Ca, seaweed, milk, and coffee intake are related to increasing hyperthyroidism risk. After conducting randomized clinical trials or prospective studies, these results can be applied to personalized nutrition to prevent or alleviate hyperthyroidism risk.

## Figures and Tables

**Table 1:** Nutrient intake and dietary patterns of the participants according to hyperthyroidism.

	No-hyperthyroidism (*n* = 38,799)	Hyperthyroidism (*n* = 530)	Adjusted OR (95% CI)^1^
Energy intake (<EER (%))	95.9 ± 0.16^2^	97.2 ± 1.07	1.088 (0.941 1.257)
CHO intake (<65 En%)	71.6 ± 0.04	72.3 ± 0.24^*∗∗∗*^	1.382 (1.108–1.723)
Protein intake (<15 En%)	13.4 ± 0.01	13.2 ± 0.09	0.909 (0.760–1.087)
Fat intake (<20 En%)	14.0 ± 0.03	13.5 ± 0.182^*∗∗*^	0.811 (0.638–1.030)
Fiber intake (<5 g)	5.62 ± 0.01	5.75 ± 0.08	1.072 (0.846–1.222)
Ca intake (<500 mg/d)	441 ± 1.15	445 ± 7.74	1.169 (1.005–1.358)
Na intake (2300 mg/d)	1384 ± 3.53	1401 ± 2.39	1.095 (0.939–1.278)
Seaweed intake (<2.6 g/day)	1.94 ± 0.01	1.99 ± 0.07	1.172 (0.995–1.375)
V–C intake (<100 mg/d)	103 ± 0.33	107 ± 2.26	1.061 (0.908–1.239)
Dietary inflammation index (<10.0)	1919 ± 7.52	1973 ± 51	1.053 (0.886–1.252)
Traditional-balanced diet^4^	25295 (65.2)^3^	372 (70.2)	1.144 (0.969–1.351)
Western-style diet^4^	26215 (67.6)	309 (58.4)^*∗∗*^	0.818 (0.699–0.957)
Rice-based diet^4^	19989 (51.5)	258 (48.6)	0.958 (0.824–1.115)
Coffee intake (number (%))			
Low (<3 C/week)	14,014 (36.1)	222 (41.8)^*∗∗*^	1
Medium (3–10 C/week)	24,414 (62.9)	306 (57.7)	0.901 (0.777–1.045)
High (≥10 C/week)	375 (0.97)	3 (0.59)	0.626 (0.231–1.697)
Alcohol intake (number, (%))			
No	21288 (54.9)	373 (70.4)^*∗∗∗*^	1
Mild drink (0–20 g)	858 (2.21)	12 (2.26)	0.761 (0.467–1.240)
Moderate drink (≥20 g)	16657 (42.9)	145 (27.3)	0.749 (0.631–0.891)
Exercise^5^ (yes; number, (%))	21,173(54.8)	292 (55.1)	1.019 (0.882–1.177)

^1^ Adjusted odds ratio (ORs) after adjusting for covariates including age, gender, residence areas, initial menstruation age, menopause, pregnancy experience, income, education, energy intake, smoking status, physical activity, WBC counts, alcohol intake, autoimmune diseases, including asthma, rheumatoid arthritis, and allergy, seaweed intake, and survey year using logistic regression models. The values represent adjusted means ± standard errors^2^ for continuous variables or number (percentage) of the categorical variables^3^. The cutoff points: ^5^ <75th percentile intake of each dietary pattern and ^5^ physical exercises with moderate activity (3 times a week).^*∗*^ Significant difference from the no-hyperthyroidism group (control) at *P* < 0.05, ^*∗∗*^*P* < 0.01, ^*∗∗∗*^*P* < 0.001.

**Table 2:** Socioeconomic and metabolic characteristics of the participants according to hyperthyroidism.

	No hyperthyroidism (*n* = 38,799)	Hyperthyroidism (*n* = 530)	Adjusted ORs (95% CI)^15^
Age^1^ (years)	53.7 ± 0.04	54.6 ± 0.25^*∗∗∗*^	1.243 (0.967–1.599)
Age during diagnosis^2^ (years)	—	47.7 ± 0.89	
Gender (men: *N*, (%))	13,653 (35.2)	70 (13.2)^*∗∗∗*^	3.011(2.253–4.023)
Metabolic syndrome (*N*, (%))	5,471 (14.1)	72 (13.6)	0.919 (0.733–1.152)
BMI^5^ (kg/m^2^)^5^	23.9 ± 0.01	23.4 ± 0.10^*∗∗*^	0.776 (0.658–0.911)
Waist circumference^s6^	80.5 ± 0.04	80.0 ± 0.27	0.980 (0.813–1.171)
Plasma total cholesterol^7^ (mg/dL)	198 ± 0.19	193 ± 1.27^*∗*^	0.866 (0.728–1.015)
Plasma HDL^8^ (mg/dL)	54.5 ± 0.07	54.0 ± 0.45	1.004 (0.856–1.178)
Plasma triglyceride^9^ (mg/dL)	126 ± 0.43	123 ± 2.84	0.918 (0.775–1.085)
Hypertension^10^ (*N*, (%))	9,506 (24.5)	125 (23.6)	1.029 (0.860–1.239)
Type 2 diabetes^11^ (*N*, (%))	9,968 (25.7)	202 (24.0)	1.107 (0.931–1.324)
Thyroid cancer (*N*, (%))	321 (0.85)	41 (7.8)^*∗∗∗*^	2.915 (1.905–4.467)
WBC counts^12^(10^9^/L)	5.71 ± 0.01	5.67 ± 0.05	0.898 (0.771–1.036)
Plasma hs-CRP^13^ (ng/mL)	0.14 ± 0.002	0.17 ± 0.02^*∗*^	0.879 (0.563–1.37)
Education^14^ (number, (%))			
<High school	6,945 (17.9)	110 (20.5)	1
High school, college	8,574(22.1)	110 (20.5)	0.863 (0.681–1.094)
College and more	23,279 (60.0)	310 (58.5)	0.863 (0.681 1.094)

The values represent adjusted means ± standard errors or number (percentage) of the subjects. The cutoff points of the reference were as follows: <55 years for age; 50 years for hyperthyroidism diagnosed age; <15 years old for initial menstruation age; <50 years old for menopause age; < 25 kg/m^2^ body mass index (BMI); < 90 cm for men and 85 cm for women waist circumferences; <230 mg/dL plasma total cholesterol concentrations; >40 mg/dL for men and 50 mg/dL for women plasma HDL cholesterol; <150 mg/dL plasma triglyceride concentrations; <140 mmHg SBP, 90 mmHg DBP plus hypertension medication; <126 ml/dL fasting serum glucose plus diabetic drug intake; <4 × 109/L white blood cell (WBC) counts; <0.5 mg/dL serum high-sensitivity C-reactive protein (hs-CRP) concentrations; high school graduation; adjusted odds ratio (ORs) and 95% confidence intervals (CI) after adjusting for covariates, including age, gender, residence areas, initial menstruation age, menopause, pregnancy experience, income, education, energy intake, seaweed intake, smoking status, physical activity, WBC counts, alcohol intake, autoimmune diseases including asthma, rheumatoid arthritis, and allergy, and survey year.

**Table 3:** Characteristics of the ten genetic variants of genes in hyperthyroidism used for the generalized multifactor dimensionality reduction analysis.

*Chr* ^*1*^	SNP^2^	Position	Mi^3^	Ma^4^	OR^5^	*P* value for ORs^6^	MAF^7^	*P* value for HWE^8^	Gene	Functional consequence
*2*	rs1881145	12634278	T	A	0.90 (0.85–0.96)	7.48.E-04	0.3495	0.8488	*TRIB2*	Intron
*2*	rs231775	204732714	A	G	0.88 (0.82–0.94)	5.54.E-05	0.2938	0.7959	*CTLA4*	Intron
*3*	rs6780858	188132110	G	A	0.90 (0.85–0.96)	6.01.E-04	0.365	0.5449	*LPP*	Intron
*6*	rs1304322089	30990958	T	C	1.18 (1.10–1.26)	5.08.E-06	0.1805	0.4865	*MUC22*	Intron
*6*	rs1800610	31543827	A	G	1.21 (1.13–1.30)	9.51.E-08	0.1921	0.6802	*TNF*	Intron
*6*	rs767861647	29976789	C	T	1.13 (1.06–1.20)	1.02.E-04	0.3008	0.486	*HLA-J*	Intron
*6*	rs3117138	32306970	C	A	1.28 (1.18–1.39)	4.69.E-09	0.1163	0.4574	*TSBP1*	Intron
*6*	rs79142022	32806673	C	T	1.56 (1.29–1.88)	4.06.E-06	0.0162	0.06767	*TAP2*	Upstream
*6*	rs78117616	33603142	C	G	1.60 (1.29–2.0)	2.69.E-05	0.0121	0.601	*ITPR3*	Intron
*8*	rs7002063	31803534	A	G	1.15 (1.08–1.22)	1.41E-05	0.270	0.516	*NRG1*	Upstream

^1^ Chromosome; ^2^ single nucleotide polymorphism; ^3^ minor alleles; ^4^ major alleles; ^5^ odds ratio (OR) and 95% confidence intervals; ^6^*P* value for OR after adjusting for age, gender, residence area, survey year, body mass index, daily energy intake, education, and income; ^7^ minor allele frequency; and ^8^ Hardy–Weinberg equilibrium. *TRIB2*, tribbles pseudokinase 2; *CTLA4*, cytotoxic T-lymphocyte-associated antigen 4; *LPP*, lipoma-preferred partner; *MUC22*, mucin 22; *TNF*, tumor necrosis factor; *HLA-J*, human leukocyte antigen, class J; *TSBP1*, testis-expressed basic protein 1; *TAP2*, transporter 2, ATP binding cassette subfamily B member; *ITPR3*, inositol 1,4,5-trisphosphate receptor type 3; and *NRG1*, neuregulin 1.

**Table 4:** Generalized multifactor dimensionality reduction (GMDR) of genetic variant-genetic variant interaction of genes related to inflammation and immunity for hyperthyroidism risk.

GMDR	Adjusted for sex and age				Adjusted for sex, age, seaweed, and BMI			
Model	TRBA^1^	TEBA^2^	*P* value^3^	CVC^5^	TRBA	TEBA	*P* value^4^	CVC
*TNF*_rs1800610	0.5243	0.5206	10 (0.0010)	9/10	0.5249	0.5211	10 (0.0010)	9/10
*MUC22*_rs1304322089 plus model 1	0.5314	0.5264	10 (0.0010)	6/10	0.5317	0.5213	10 (0.0010)	4/10
*MIR3681*_rs1881145 plus model 2	0.5388	0.5224	9 (0.0107)	7/10	0.5391	0.5185	9 (0.0107)	4/10
*MIR3681*_rs1881145 *CTLA4*_rs231775 *LPP*_rs6780858 *HLA-J*_rs767861647	0.5465	0.5137	9 (0.0107)	3/10	0.5473	0.5186	9 (0.0107)	4/10
*TNF*_rs1800610 plus model 4	0.5622	0.5181	9 (0.0107)	8/10	0.5624	0.5205	9 (0.0107)	8/10
***CTLA4*_rs231775 *LPP*_rs6780858 *HLA-J*_rs767861647 plus model 3**	**0.5880**	**0.5205**	**9 (0.0107)**	**10/10**	**0.5888**	**0.5221**	**10 (0.0010)**	**10/10**
***TSBP1*_rs3117138 plus model 6**	**0.6164**	**0.5168**	**9 (0.0107)**	**10/10**	**0.6172**	**0.5193**	**9 (0.0107)**	**10/10**
*NRG1*_rs7002063 plus model 7	0.6454	0.5211	8 (0.0547)	10/10	0.6462	0.5221	10 (0.0010)	10/10
*TSBP1*_rs3117138 *ITPR3*_rs78117616 plus model 7	0.6515	0.5158	8 (0.0547)	7/10	0.6522	0.5193	9 (0.0107)	6/10
*TAP2*_rs79142022 plus model 9	0.6550	0.5176	9 (0.0107)	10/10	0.6558	0.5228	9 (0.0107)	10/10

^1^ Trained balanced accuracy; ^2^ test balance accuracy; ^3,4^*P* value for the significance of GMDR model by sign test. ^3^ without and ^4^ with adjusting for covariates; ^5^ crossvalidation consistency; and BMI, body mass index.

**Table 5:** Adjusted odds ratio and 95% confidence intervals for hyperthyroidism by the PRS with 7 SNPs after covariate adjustments according to age, gender, metabolic syndrome, and nutrient intake.

	Low PRS (*N* = 15,403)	Medium PRS (*N* = 20,929)	High PRS (*N* = 2,997)	Gene-nutrient interaction *P* value
Less aged people^1^	1	1.434 (1.152–1.786)	2.850 (1.771–4.588)	0.0366
More aged people		1.135 (0.572–2.255)	1.135 (0.572–2.255)	
Men	1	1.172 (0.773–1.778)	2.856 (1.187–6.871)	0.3095
Women		1.276 (1.080–1.507)	1.811 (1.176–2.787)	
Without MetS	1	1.338 (1.130–1.583)	2.278 (1.530–3.391)	0.0165
With MetS	1	0.882 (0.590–1.319)	0.352 (0.048–2.590)	
Low BMI^2^	1	1.345 (1.121–1.613)	2.170 (1.402–3.359)	0.1745
High BMI		1.046 (0.777–1.409)	1.389 (0.596–3.236)	
Low energy intake^3^	1	1.199 (0.975–1.474)	2.740 (1.742–4.310)	0.0084
High energy intake	1	1.350 (0.997–1.719)	1.027 (0.474–2.229)	
Low CHO^4^	1	1.561 (1.174–2.077)	2.573 (1.376–4.813)	0.4478
High CHO		1.153 (0.958–1.387)	1.720 (1.048–2.823)	
Low protein^5^	1	1.144 (0.926–1.414)	1.983 (1.168–3.370)	0.4287
High protein		1.415 (1.126–1.778)	1.960 (1.111–3.456)	
Low fat^6^	1	1.114 (0.924–1.343)	2.021 (1.271–3.215)	0.0972
High fat		1.639 (1.240–2.167)	1.885 (0.932–3.815)	
Low fiber^7^	1	1.261 (1.057–1.505)	2.126 (1.388–3.255)	0.7071
High fiber		1.418 (0.559–3.593)	1.265 (0.915–1.749)	
Low Ca^8^	1	1.192 (0.960–1.480)	2.835 (1.764–4.555)	0.0131
High Ca		1.333 (0.999–1.715)	1.115 (0.561–2.218)	
Low seaweed^9^	1	1.276 (0.939–1.733)	3.433 (1.881–6.265)	0.0201
High seaweed		1.262 (1.081–1.473)	1.956 (1.328–2.880)	
Low vegetables^10^	1	1.302 (1.090–1.555)	2.195 (1.432–3.365)	0.7468
High vegetables		1.133 (0.826–1.554)	1.252 (0.496–3.160)	
Low fruits^11^	1	1.211 (1.011–1.451)	2.186 (1.415–3.377)	0.2953
High fruits		1.425 (1.052–1.930)	1.412 (0.602–3.313)	
Low milk^12^	1	1.221 (1.008–1.479)	2.908 (1.923–4.396)	<0.0001
High milk		1.346 (1.034–1.752)	0.343 (0.084–1.401)	
Low DII^13^	1	1.381 (1.018–1.873)	3.451 (1.823–6.533)	0.0499
High DII		1.225 (0.998–1.456)	1.514 (0.925–2.477)	
Low coffee^14^	1	1.053 (0.829–1.336)	2.443 (1.421–4.201)	0.0189
High coffee		1.431 (1.167–1.756)	1.615 (0.925–2.823)	
Low alcohol^15^	1	1.291 (1.075–1.550)	1.681 (1.024–2.758)	0.3348
High alcohol		1.184 (0.884–1.585)	2.567 (1.375–4.791)	
Nonsmoking	1	1.305 (1.104–1.541)	1.836 (1.193–2.825)	0.1947
Former + current smoking		1.020 (0.668–1.558)	2.700 (1.120–6.513)	
No exercise^16^	1	1.361 (1.082–1.712)	2.025 (1.127–3.641)	0.6968
Exercise		1.198 (0.972–1.477)	1.893 (1.131–3.169)	
Low KBD^17^	1	1.262 (1.081–1.473)	1.956 (1.328–2.880)	0.7043
High KBD		1.216 (1.006–1.469)	1.960 (1.220–3.147)	
Low WSD^17^	1	1.227 (1.056–1.426)	1.830 (1.254–2.673)	0.8925
High WSD		1.234 (1.030–1.477)	1.774 (1.107–2.842)	
Low RBD^17^	1	1.202 (0.998–1.493)	1.806 (1.208–2.650)	0.0638
High RBD		1.117 (0.930–1.341)	1.558 (0.951–2.552)	

Values represent the odds ratio and 95% confidence intervals. PRS with 7 SNPs was divided into three categories (0–4, 5–7, and ≥8) by tertiles as the low, medium, and high PRS groups of the best model of GMDR. The cutoff points were as follows: ^1^ <55 years old, ^2^ <25 kg/m2 BMI, ^3^ <estimated energy intake, ^4^ < 70% carbohydrate (CHO) intake, ^5^ < 13% protein intake, ^6^ < 15% fat intake, ^7^ < 5 g/d fiber intake, ^8^ < 500 mg/d Ca intake, ^9^ <2.65 g/day seaweed intake, ^10^ < 160 g/d vegetable intake, ^11^ < 82 g/d fruits intake, ^12^ < 150 ml/d milk, ^13^ < 10.0 scores of dietary inflammation index (DII), ^14^ < 3 cup/week coffee intake, ^15^ < 20 g/d alcohol intake, ^16^ < moderate exercise 30 min/d for 3 times/week, and ^17^ <75^th^ percentile of each dietary pattern. Multiple logistic regression models include the corresponding main effects, interaction terms of SNPs and main effects (energy and nutrient intake), and potential confounders such as age, gender, residence areas, initial menstruation age, menopause, pregnancy experience, income, education, energy intake, seaweed intake, smoking status, physical activity, WBC count, alcohol intake, autoimmune diseases including asthma and allergy, hyperthyroid treatments, and survey year. Multiple logistic regression models include the corresponding main effects, interaction terms of SNPs and main effects (energy and nutrient intake), and potential confounders. Reference was the low-PRS. KBD, Korean balanced diet intake; WSD, Western-style diet intake; and RBD, rice-based diet intake.

## Data Availability

The data presented in this study are available on request from the corresponding author.

## Abbreviations

PRS: Polygenic risk scores
WSD: Western-style diet
*T4*: Tetraiodothyronine
*T3*: Triiodothyronine
*TSH*: Thyroid-stimulating hormone
*HLA*: Human leukocyte antigen
*CD152*: Cluster of differentiation 152
*PTPN22*: Protein tyrosine phosphatase nonreceptor type 22
*MIR3681*: MicroRNA 36891
*CTLA4*: Cytotoxic T-lymphocyte-associated antigen 4
*LPP*: Lipoma-preferred partner
*MUC22*: Mucin 22
*LPP*: Lipoma-preferred partner
*IL*: Interleukin
*IL2RA*: IL-2 receptor alpha chain
*IL23R*: IL-23 receptor
*FCRL3*: Fc receptor-like 3
*TNF-α*: Tumor necrosis factor-alpha
BMI: Body mass index
WBC: White blood cell
KoGES: Korean genome and epidemiology study
WBC: White blood cell
SQFFQ: Semiquantitative food frequency questionnaire
DII: Dietary inflammatory index
SNP: Single nucleotide polymorphism
HWE: Hardy–Weinberg equilibrium
GMDR: Generalized multifactor dimensionality reduction
GWAS: Genome-wide association study
LD: Linkage disequilibrium
TRBA: Trained balanced accuracy
TEBA: Test balance accuracy
CVC: Crossvalidation consistency
PRSs: Polygenetic risk scores
MetS: Metabolic syndrome
ORs: Odds ratio
Cis: Confidence intervals
ANCOVA: Analysis of covariance
hs-CRP: High-sensitivity C-reactive protein
KBD: Korean balanced diet
WSD: Western-style diet
RBD: Rice-based diet.

## Additional Points

The study was conducted according to the Declaration of Helsinki's guidelines and approved by the Institutional review board of the Korean National Institute of Health (KBP-2015-055). We additionally obtained approval from the Institutional Review Board of Hoseo University (1041231-150811-HR-034-01).

## Supplementary Materials

Supplementary Materials This study included a supplemental table and figures.
