# Supplementary material for: Association of Polygenetic Risk Scores Related to Immunity and Inflammation with Hyperthyroidism Risk and Interactions between the Polygenetic Scores and Dietary Factors in a Large Cohort
Source: J Thyroid Res. 2021 Sep 14;2021:7664641. doi: 10.1155/2021/7664641 (PMC8457978; doi:10.1155/2021/7664641)
Supplement: Supplementary Materials — This study included a supplemental table and figures. [file 7664641.f1.zip › 7664641.f1/Supplemental figure legends.docx]

Supplemental figure.

Figure S1. Frequencies of hyperthyroidism among participants in the low-, medium- and high-PRS groups (determined using the 7-SNP genetic variant interaction model).

A. Participants categorized by age.

B. Participants categorized by metabolic syndrome.

C. Participants categorized by energy intake (cutoff value: 65%/day).

D. Participants categorized by calcium intake (cutoff value: 500 mg/day).

E. Participants categorized by milk intake (cutoff value: 150 ml/day).

F. Participants categorized by seaweed intake (cutoff value: 2.65g/day).

G. Participants categorized by coffee intake (cutoff value: 2.65g/day).

H. Participants categorized by dietary inflammation index (DII) scores (cutoff value: 75^th^ percentile).

PRS with 7 SNPs, the best model of GMDR, was divided into 3 categories (0-4, 5-7, and ≥8) by tertiles as the low-PRS, medium-PRS, and high-PRS groups. The nutrient and diet variables were categorized into two groups based on the specified cutoff values. The frequencies of thyroid cancer of PRS groups were calculated in low and high intake groups. Non-MetS, having not metabolic syndrome; MetS, having metabolic syndrome; EER, estimated energy intake

^**^ Significantly different between the low- and high-PRS groups by χ2 test at P<0.01. ^***^ at P<0.001.
