# Supplementary material for: Association of Polygenetic Risk Scores Related to Immunity and Inflammation with Hyperthyroidism Risk and Interactions between the Polygenetic Scores and Dietary Factors in a Large Cohort
Source: J Thyroid Res. 2021 Sep 14;2021:7664641. doi: 10.1155/2021/7664641 (PMC8457978; doi:10.1155/2021/7664641)
Supplement: Supplementary Materials — This study included a supplemental table and figures. [file 7664641.f1.zip › 7664641.f1/Supplemental Table 1.docx]

Supplemental Table 1. Factor loadings of food groups in dietary patterns identified using principle component analysis

|  | Traditional balanced diet | Western-style diet | Rice-main diet |
| --- | --- | --- | --- |
| Rice | -7 | 11 | 92 |
| Grains | 6 | -6 | -90 |
| Noodles | -2 | 60 | 0 |
| Breads | 8 | 37 | -6 |
| Cakes | 9 | 37 | -4 |
| Cookies | 53 | 5 | -4 |
| Beans | 47 | 8 | -7 |
| Tubers | 41 | 2 | 4 |
| Kimchi | 27 | 22 | -5 |
| Eggs | 0 | 73 | -9 |
| Fast foods | 78 | 3 | 1 |
| Green vegetables | 73 | 7 | 6 |
| Mushrooms | 60 | -2 | -2 |
| White vegetables | 55 | 17 | 1 |
| Fatty fishes | 63 | 17 | 5 |
| White fishes | 42 | 21 | 6 |
| Crabs | 23 | 9 | -2 |
| Processed meats | 29 | 46 | 10 |
| Red meats | 10 | 65 | -6 |
| Soups | 21 | 43 | 4 |
| Chicken | 59 | 2 | -5 |
| Seaweed | 37 | 7 | -7 |
| Milk and its products | 41 | 8 | -1 |
| Beverages | 4 | 24 | 12 |
| Coffee | 4 | 27 | 13 |
| Teas | 43 | -3 | -10 |
| Fruits | 40 | 7 | 8 |
| Korean fermented foods | -3 | 18 | 10 |
| Alcohols | 26 | 9 | -14 |
| Nuts | 0.25 | 0.37 | -0.19 |
| Variance explained by each factor | 4.417 | 2.357 | 1.780 |

Values are multiplied by 100 and rounded to the nearest integer. Values greater than 0.4 represented higher consumption in the diet pattern.
